# New Perspectives on Risk Assessment and Anticoagulation in Elective Spine Surgery Patients: The Impact of Ultra-Minimally Invasive Endoscopic Surgery Techniques on Patients with Cardiac Disease

**DOI:** 10.3390/jpm14070761

**Published:** 2024-07-17

**Authors:** Alexandre Siciliano, Kai-Uwe Lewandrowski, Sergio Luis Schmidt, Rossano Kepler Alvim Fiorelli, Paulo Sérgio Teixeira de Carvalho, Abduljabbar Alhammoud, Stenio Karlos Alvim Fiorelli, Marcos Arêas Marques, Morgan P. Lorio

**Affiliations:** 1 Department of General and Specialized Surgery, Gaffrée e Guinle Universitary Hospital, Federal University of the State of Rio de Janeiro (UNIRIO), Rio de Janeiro 22290-240, Brazil; alexandre.siciliano@unirio.br (A.S.); rossano.fiorelli@unirio.br (R.K.A.F.); 2 Center for Advanced Spine Care of Southern Arizona, Tucson, AZ 85712, USA; 3 Department of Neurology, Federal University of the State of Rio de Janeiro (UNIRIO), Rio de Janeiro 21941-901, Brazil; slschmidt@terra.com.br; 4 Pain and Spine Minimally Invasive Surgery Service at Gaffre Guinle University Hospital, Rio de Janeiro 20270-004, Brazil; paulo.carvalho@unirio.br; 5 Department of Orthopaedic Surgery, University of Arizona College of Medicine—Tucson Campus, Health Sciences Innovation Building (HSIB), 1501 N. Campbell Avenue, Tower 4, 8th Floor, Suite 8401, Tucson, AZ 85721, USA; aalhammoud@arizona.edu; 6 Chefe do Serviço de Angiologia e Cirurgia Vascular do Hospital Universitario Gaffrée e Guinle, Universidade Federal do Estado do Rio de Janeiro (UNIRIO), Rio de Janeiro 22290-250, Brazil; stenio.fiorelli@unirio.br; 7 Serviço de Angiologia e Cirurgia Vascular, Hospital Universitário Gaffrée e Guinle, Universidade Federal do Estado do Rio de Janeiro (UNIRIO), Rio de Janeiro 21941-909, Brazil; marcos.areas@ebserh.gov.br; 8 Hospital Universitário Pedro Ernesto da Universidade do Estado do Rio de Janeiro (UERJ), Rio de Janeiro 20550-170, Brazil; 9 Advanced Orthopedics, 499 East Central Parkway, Altamonte Springs, FL 32701, USA; drlorio@advancedhere.com

**Keywords:** minimally invasive spinal surgery, endoscopy, preoperative cardiac clearance, anticoagulation, echocardiography, laboratory studies

## Abstract

The advent of ultra-minimally invasive endoscopic spine surgery, characterized by significantly reduced surgery times, minimal blood loss, and minimal tissue trauma, has precipitated a paradigm shift in the preoperative management of patients with cardiac disease undergoing elective spine procedures. This perspective article explores how these advancements have influenced the requirements for preoperative cardiac workups and the protocols surrounding the cessation of anticoagulation and antiplatelet therapies. Traditionally, extensive cardiac evaluations and the need to stop anticoagulation and antiplatelet agents have posed challenges, increasing the risk of cardiac events and delaying surgical interventions. However, the reduced invasiveness of endoscopic spine surgery presents a safer profile for patients with cardiac comorbidities, potentially minimizing the necessity for rigorous cardiac clearance and allowing for more flexible anticoagulation management. This perspective article synthesizes current research and clinical practices to provide a comprehensive overview of these evolving protocols. It also discusses the implications of these changes for patient safety, surgical outcomes, and overall healthcare efficiency. Finally, the article suggests directions for future research, emphasizing the need for updated guidelines that reflect the reduced perioperative risk associated with these innovative surgical techniques. This discussion is pivotal for primary care physicians, surgeons, cardiologists, and the broader medical community in optimizing care for this high-risk patient population.

## 1. Introduction

The management of patients with cardiac disease requiring traditional spine surgery presents a multifaceted clinical dilemma, necessitating a careful balance between the urgency of spinal intervention and the risks of cardiac complications. This challenge is compounded by the need for preoperative cardiac clearance, anticoagulation and antiplatelet agent management, deep vein thrombosis (DVT) prophylaxis, and vigilant postoperative surveillance for cardiac events.

Cardiac complications are recognized as a significant risk factor for adverse outcomes in spine surgery. Patients with existing cardiac conditions, particularly those with a history of coronary artery disease, heart failure, or arrhythmias, are at an elevated risk of perioperative cardiac events [1]. The decision to proceed with surgery must, therefore, be predicated upon a thorough preoperative cardiac evaluation. Guidelines (Table 1), such as those proposed by the American College of Cardiology/American Heart Association (ACC/AHA) [1] and the European Society of Cardiology [2], offer the criteria for cardiac risk assessment and management in patients undergoing non-cardiac surgery [3,4]. These criteria emphasize the importance of stratifying patients based on their clinical risk factors and the anticipated surgical stress.

Anticoagulation management poses another significant challenge. Patients on long-term anticoagulation therapy, such as those with mechanical heart valves or atrial fibrillation, require a carefully orchestrated plan for perioperative anticoagulation [5]. The risk of thromboembolism must be weighed against the risk of surgical bleeding [6]. Bridging therapy, involving the temporary substitution of long-term anticoagulants with short-acting agents, is often employed, although its benefits must be carefully weighed against potential risks [7]. Similarly, DVT prophylaxis is critical in spine surgery due to the associated risk of venous thromboembolism (VTE) if there is prolonged immobilization and intraoperative positioning [8,9,10,11,12]. The choice of prophylactic agent, whether it be pharmacological or mechanical, is determined by the patient’s overall VTE risk and must be balanced with the risk of postoperative bleeding [8,13,14,15].

Antiplatelet therapy is widely used for the secondary prevention of stroke, myocardial infarction (MI), and peripheral arterial disease, with such patients typically receiving single antiplatelet therapy with acetylsalicylic acid (ASA) or dual antiplatelet therapy with ASA and a P2Y12 inhibitor such as clopidogrel or ticagrelor. The premature discontinuation of antiplatelet therapy has the potential to expose patients to an increased risk of perioperative acute coronary syndromes, with stent thrombosis being most feared because of its high associated morbidity and mortality. In contrast, the continuation of antiplatelet therapy without perioperative interruption exposes patients to an increased risk of bleeding, which, in turn, can increase the risk of adverse cardiovascular (CV) events. Therefore, the perioperative management of antiplatelet therapy requires balancing the risk of CV or thrombotic events against the risk of bleeding, the latter of which is driven by the surgery/procedure-related bleeding risk [16].

Preoperative workup and postoperative surveillance for cardiac events is an integral component of the care plan for these patients. This involves monitoring for signs of myocardial infarction, heart failure, and arrhythmias, which are not uncommon after major surgery [17]. The use of cardiac biomarkers, electrocardiograms [1], and clinical vigilance plays a pivotal role in the early detection and management of these events. However, the benefit of the routine use of echocardiogram in the preoperative workup of patients undergoing elective musculoskeletal surgeries as recommended according to the ACC/AHA criteria (Table 2) is yet to be demonstrated [18,19]. With traditional open surgery, the management of patients with cardiac disease undergoing traditional spine surgery requires a nuanced, multidisciplinary approach. Adherence to established guidelines for preoperative clearance, coupled with tailored strategies for anticoagulation management, DVT prophylaxis, and postoperative cardiac surveillance, is essential to optimize patient outcomes and mitigate risks.

With the advent of minimally invasive spinal surgery, and its newest version—the ultra-minimally invasive endoscopic spinal decompression surgery—these traditional preoperative cardiac clearance and management criteria are being revisited as the surgical stress from the endoscopic decompression surgery performed through a less than 1 cm incision causing negligible surgical access trauma is quite low. Many of these surgeries are being performed in an outpatient surgery center under local anesthesia and sedation and cause very little blood loss [20,21,22,23]. Thus, employing the same cardiac clearance criteria preoperatively to stratify high-risk patients, or take them off anticoagulation and/or antiplatelet agents because of higher bleeding risk seems outdated considering the short surgery times employing conscious sedation with moderate sedation/analgesia strategies with the use of fewer or lower doses of drugs. Therefore, the authors revisited some of the common controversial issues regarding the medical work-up and management of anticoagulation and antiplatelet agents and preoperative cardiac workup in the context of an ultra-minimally invasive endoscopic spinal decompression procedure to avoid unnecessary system overutilization, where the cardiac interventions dictated by the management of the underlying heart disease may be riskier than the short endoscopic lumbar decompression procedure.

## 2. Perceptions of Spinal Surgery Amongst Medical Professionals

The persistent myth that all lumbar spinal surgeries are inherently aggressive and high-risk continues to pervade the medical community, particularly among primary care and other specialty physicians [24]. This entrenched belief, while rooted in historical practices, overlooks the significant advancements in spinal surgery techniques and technologies over recent years. Modern lumbar procedures, such as minimally invasive and endoscopic techniques, have revolutionized the field, offering patients safer options with reduced complication rates, shorter recovery times, and less postoperative pain. These advancements challenge the outdated notion that lumbar spinal surgery is a last resort due to its aggressiveness and risk profile. The persistence of this myth not only undermines the rapid innovation cycle in spinal surgery [25] but also potentially deprives patients of earlier intervention options that could prevent the progression of debilitating conditions [26,27]. Patients may be deprived of access to the most appropriate and advanced spine care options available because the referring physicians may give medical advice on the basis of outdated perceptions rather than current best practices capable of improving patient functioning by providing favorable clinical outcomes with low-risk targeted lumbar decompression procedures used in the treatment of spinal stenosis and claudication.

## 3. The Paradigm Shift in Medical Necessity Criteria for Spinal Surgery

A paradigm shift is underway, steering the medical necessity criteria from a predominantly image-based approach towards a more nuanced, personalized care model centered around validated pain generators [28]. This transformation is reshaping how surgeons evaluate and manage spinal disorders, emphasizing a comprehensive understanding of the patient’s pain and functional impairment over radiographic findings alone [29,30].

Historically, the decision to proceed with spinal surgery has been heavily influenced by imaging results. Techniques such as Magnetic Resonance Imaging (MRI) and Computed Tomography (CT) scans have been pivotal in identifying and grading structural spinal abnormalities such as instability and deformity. While these imaging modalities are invaluable, their limitations in reliability in identifying spinal pain generators are becoming increasingly recognized [31]. Research has shown that many imaging-detected abnormalities may be incidental and not necessarily the source of the patient’s symptoms [32,33]. For instance, disk degeneration or herniation observed on an MRI can be asymptomatic and may not correlate with the patient’s pain or functional status [34]. The new approach to determining the necessity for spinal surgery involves a more holistic assessment of the patient [29]. This method combines the objective findings from imaging with a thorough evaluation of the patient’s symptoms, clinical history, physical examination [35], and response to diagnostic injections [36]. This patient-centered model acknowledges that pain is a subjective and complex experience influenced by biological, psychological, and social factors.

One of the cornerstones of this paradigm shift is the concept of validated pain generators [30,37,38,39,40,41]. Instead of relying solely on anatomical abnormalities, surgeons are now focusing on identifying specific structures or conditions that are most likely contributing to the patient’s pain. This could include nerve compression, discal, and facet joint pathology, ranging from cysts, tears, and tethering, among others [42,43]. Identifying these pain generators often requires a combination of diagnostic modalities, including diagnostic injections, physical examination techniques, and a detailed patient history [35,36]. This personalized approach to spinal surgery has several benefits. First, it helps in avoiding unnecessary surgeries by ensuring that the procedure addresses the actual source of pain. Second, it enhances the likelihood of successful surgical outcomes, as the interventions are more targeted and tailored to the individual’s specific condition. Third, it fosters a more collaborative patient–surgeon relationship, where shared treatment decisions are made considering the patient’s preferences, expectations, and lifestyle. Moreover, this shift has significant implications for healthcare systems and payers. By reducing the number of aggressive surgeries and their ensuing revisions prompted by complications and rapid disease progression, it can lead to cost savings and a more efficient allocation of healthcare resources. It also aligns with the broader trend in medicine towards value-based care, where the emphasis is on patient outcomes and satisfaction.

## 4. Risk–Benefit Analysis

Spinal surgery, like any surgical intervention, carries inherent risks and benefits that must be weighed carefully, especially in high-risk patients. This includes a thorough evaluation of the necessity of the surgery against potential cardiac risks. For patients with known cardiac disease, particularly those classified as high-risk (e.g., with recent cardiac events, severe valve disease, or significant arrhythmias), the decision to proceed with surgery should involve a comprehensive assessment of the potential for perioperative cardiac complications. This assessment should include a review of recent cardiac evaluations, consultations with relevant specialists, and the consideration of the urgency and expected benefits of the surgery. In some cases, non-surgical management may be a preferable alternative, or the preoperative optimization of cardiac conditions may be necessary to reduce the risk of adverse events. Risk exposure may also be mitigated by performing more targeting decompression surgeries such as the transforaminal endoscopic laminotomy, foraminotomy, and microdiscectomy.

## 5. Nuances of the ACC/AHA, ESC, and ASA Risk Factor Classification

**Figure 1:**
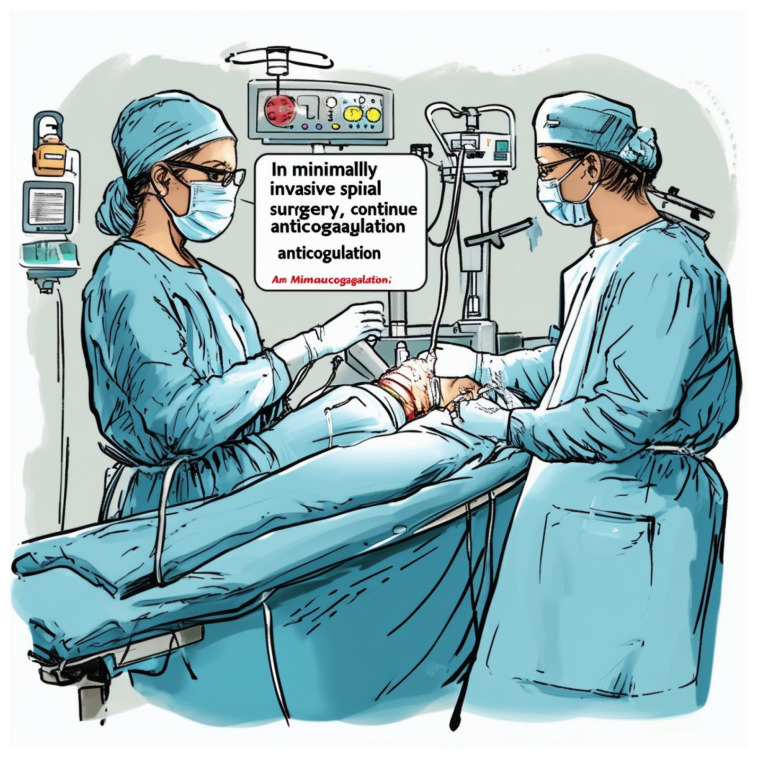
The advent of ultra-minimally invasive endoscopic spine surgery has revolutionized preoperative management in cardiac patients. This perspective article explores how these advancements have influenced preoperative cardiac workups and anticoagulation protocols, reducing surgery times, blood loss, and tissue trauma. Traditionally, extensive cardiac evaluations and anticoagulation cessation posed challenges, but the reduced invasiveness of endoscopic surgery offers a safer profile. This synthesis of research and clinical practices provides insights into patient safety, surgical outcomes, and healthcare efficiency, with implications for future guidelines and care optimization.

The American College of Cardiology/American Heart Association (ACC/AHA) cardiac risk factor classification and the American Society of Anesthesiologists (ASA) physical status classification system are both critical tools used in the preoperative assessment of patients undergoing elective spine surgery, yet they serve different, albeit sometimes overlapping, purposes (Figure 1). The ACC/AHA cardiac risk factor classification specifically focuses on evaluating the risk of cardiac complications associated with surgery. It categorizes patients based on their likelihood of experiencing adverse cardiac events during or after a surgical procedure. The ACC/AHA guidelines consider a range of cardiac-specific factors, including the presence of coronary artery disease, heart failure, valvular disease, arrhythmias, and a history of myocardial infarction, among others. The aim is to guide physicians in identifying patients at higher risk who might benefit from further cardiac evaluation or intervention before undergoing surgery.

The ASA physical status classification system, on the other hand, is broader in scope. It assesses the overall physical health and medical readiness of a patient for anesthesia and surgery, not just from a cardiac standpoint but encompassing the entire spectrum of health issues [44,45,46]. The ASA classification ranges from ASA I (a healthy patient) to ASA V (a moribund patient who is not expected to survive without the operation). This system is used to predict the perioperative risks associated with anesthesia and surgery based on the patient’s overall health status.

While both systems are used in preoperative assessment, they approach patient evaluation from different angles. The ACC/AHA classification is specifically cardiac-focused and is particularly useful for patients with known heart disease or risk factors for cardiac complications. It helps in tailoring cardiac-specific perioperative management, such as deciding on the need for preoperative cardiac stress testing or perioperative beta-blockade. The ASA classification, being more holistic, takes into account not just the cardiac status but also other comorbidities like pulmonary disease, diabetes, renal function, and even psychological factors. It is used by anesthesiologists to assess the overall risk associated with administering anesthesia and performing surgery. In practice, there can be significant overlap. For instance, a patient with severe cardiac disease (high ACC/AHA risk) is likely to have a higher ASA classification due to the increased overall perioperative risk. However, a patient could have a high ASA classification due to non-cardiac reasons (such as severe pulmonary disease) while having a low ACC/AHA risk. Both the ACC/AHA cardiac risk factor classification and the ASA physical status classification are integral to the preoperative evaluation of spine surgery patients. They serve different but complementary roles. Understanding and utilizing both classifications allow for a more comprehensive and nuanced approach to preoperative planning and risk stratification.

The European Society of Cardiology recently published guidelines on the cardiovascular assessment and management of patients undergoing non-cardiac surgery (NCS) was endorsed by the European Society of Anaesthesiology and Intensive Care (ESAIC) [2]. According to the document, cardiovascular morbidity and mortality in patients undergoing NCS are determined by two main factors: patient-related risk and the type of surgery or procedure, including the circumstances under which it takes place (experience of institution and elective vs. emergency procedure). The type of anesthesia and anesthetic drugs may also influence the risk of complications in patients at intermediate to high cardiac risk undergoing NCS. The risk may be reduced by an adequate preoperative evaluation and the proper selection of the type and timing of the surgical procedure.

## 6. The Kitchen Sink Approach

The “kitchen sink” approach to preoperative clearance for patients with cardiac disease scheduled for elective joint replacement or spinal surgery has become a subject of critical reassessment. This method, characterized by an exhaustive battery of lab tests, imaging, and functional assessments, is often implemented with the intention of thoroughness. However, it increasingly appears to be not only superfluous but potentially detrimental, failing to meaningfully alter management strategies or reduce surgical risks. Traditionally, a comprehensive preoperative workup is considered a prudent step, particularly for high-risk patients with known cardiac issues. The rationale is rooted in caution—to preemptively identify and mitigate any potential intraoperative and postoperative complications. However, this approach often leads to an extensive array of diagnostic tests, including complete blood counts, electrolyte panels, coagulation profiles, cardiac stress tests, echocardiograms, and even pulmonary function tests, regardless of the patient’s specific history or the inherent risks of the planned procedure.

The drawbacks of this indiscriminate screening are manifold. First, it leads to a significant increase in healthcare costs without corresponding benefits. Many of these tests, especially advanced cardiac imaging and functional assessments, raise healthcare expenditure. Yet, studies indicate that they rarely result in changes to surgical plans or improve outcomes for patients undergoing routine elective surgeries like joint replacements or uncomplicated spinal procedures [4,18,19]. Moreover, the over-reliance on preoperative testing can inadvertently cause harm. False positives, a not uncommon outcome of extensive testing, can lead to unnecessary anxiety, further invasive testing, and even unwarranted medical interventions, each carrying its own risks [47,48,49]. This cascade effect not only burdens the patient but can also delay or outright cancel the elective surgery, thereby prolonging pain and disability for the patient.

Another significant concern is the barrier this “kitchen sink” approach creates to accessing surgical care. The lengthy process of undergoing multiple preoperative tests can be daunting, particularly for elderly or frail patients, or those with limited access to healthcare resources. In some cases, patients may opt to forego surgery altogether rather than navigate this labyrinthine preoperative pathway. There is a growing consensus that a more tailored approach to preoperative assessment is needed. Such an approach would consider the individual patient’s cardiac history, the nature of the elective surgery, and current best practices. For many patients, a detailed history and physical examination, along with basic laboratory tests, are sufficient to assess surgical risk. Additional tests should be reserved for those with specific indications, such as recent changes in cardiac symptoms or a history of significant cardiovascular disease.

## 7. Tailoring to Cardiac Risk Categories

The preoperative cardiac evaluation for endoscopic lumbar spinal decompression surgery should be individualized based on the patient’s cardiac risk category. This approach ensures a balance between thoroughness and avoiding unnecessary testing, ultimately enhancing patient safety and surgical outcomes. The current ACC/AHA guidelines provide a framework for stratifying patients into high (>5%), intermediate (1–5%), and low (<1%) cardiac risk categories (Table 1).

High Cardiac Risk (>5%): Patients in this category often have a history of significant cardiovascular conditions such as recent myocardial infarction, unstable angina, or severe valvular disease. For these patients, a comprehensive preoperative cardiac evaluation is crucial. The ACC/AHA recommends the following:

1. Cardiac Stress Testing: If the patient has not undergone recent testing and is capable of physical exertion, a stress test can assess the risk of ischemic cardiac events.
2. Echocardiography: Useful in evaluating left ventricular function and structural heart disease, particularly if there is a history of heart failure or valvular pathology.
3. Electrocardiogram (ECG): Essential for all high-risk patients to check for arrhythmias, ischemic changes, or other abnormalities.
4. Cardiology Consultation: Consider a preoperative consultation with a cardiologist to optimize the management of identified cardiac conditions.

Intermediate Cardiac Risk (1–5%): Patients in this group may have stable angina, a history of myocardial infarction, compensated heart failure, diabetes, or renal insufficiency. The focus here is on identifying potential risk factors that could impact patient outcomes during or after surgery.

1. Baseline ECG: Recommended for all intermediate-risk patients, particularly if they have a history of heart disease or are symptomatic.
2. Functional Capacity Assessment: Assessing the patient’s ability to perform activities of daily living can help gauge cardiac reserve.
3. Selective Cardiac Testing: Based on patient history and symptoms, additional tests like echocardiography or non-invasive stress testing may be warranted.

Low Cardiac Risk (<1%): For patients with low cardiac risk undergoing endoscopic lumbar decompression, extensive cardiac testing is usually not indicated unless specific cardiac symptoms or history suggest otherwise. The following basic assessment protocol often suffices:

1. Clinical Evaluation: A thorough history and physical examination are often sufficient. Focus on any symptoms of undiagnosed cardiac conditions.
2. Basic Laboratory Tests: Basic tests including complete blood count, electrolytes, and renal function tests are advised to rule out any factors that could complicate anesthesia or surgery.
3. ECG: Consider for patients with a history of cardiovascular disease, even if they fall into the low-risk category.

The most recent guidelines from the European Society of Cardiology and the consensus among European surgical associations consider the elective ultra-minimally invasive endoscopic spine surgical procedure as a low-risk procedure (<1%) [2,50]. Regardless patient’s age, related cardiovascular risk factors, or established cardiovascular disease, no further testing other than accurate history and clinical examination and standard lab tests would be recommended preoperatively. Advise on stopping smoking and optimizing guideline-recommended medical therapy is warranted [2].

## 8. Anticoagulation and DVT Prophylaxis

One of the critical steps in the preoperative preparation of patients undergoing spinal surgery is the meticulous review and management of their medications, particularly focusing on anticoagulant and antiplatelet therapy to strike a balance between reducing thrombotic risks and preventing surgical bleeding. Coordination with the patient’s cardiologist or primary care physician is essential to tailor a plan that minimizes the risk of bleeding while safeguarding against thrombotic events, especially in those with cardiovascular comorbidities. For patients on long-term anticoagulation for conditions like atrial fibrillation or mechanical heart valves, bridging therapy or the temporary cessation of these medications might be considered based on individual thrombotic and hemorrhagic risk profiles. Similarly, the management of antiplatelet agents, often prescribed for coronary artery disease, requires careful consideration. Decisions regarding the continuation, modification, or temporary withdrawal of these agents should be based on a thorough assessment of the patient’s cardiovascular history, the type of spinal procedure planned, and the specific pharmacological properties of the medications involved.

Patients undergoing spinal surgery are at an increased risk of VTE, which includes DVT and pulmonary embolism (PE) [4,6,8,12]. The implementation of appropriate VTE prophylaxis is thus a critical component of preoperative planning. The choice of prophylaxis should be individualized based on the patient’s risk factors for both VTE and bleeding [4]. Standard prophylactic measures include pharmacologic agents such as low-molecular-weight heparin (LMWH), unfractionated heparin (UFH), or direct oral anticoagulants (DOACs), and/or mechanical methods such as graduated compression stockings or intermittent pneumatic compression devices. The timing of the initiation and duration of prophylaxis should be aligned with the patient’s overall risk profile and the specifics of the spinal procedure. For patients with significant bleeding risks or those on long-term anticoagulation, the strategy may need to be modified to balance the risks of thrombosis and bleeding. Additionally, early mobilization post-surgery is encouraged as part of VTE prevention.

Modern oral drugs for anticoagulation and DVT prophylaxis represent significant advancements in the management of thromboembolic disorders. These oral anticoagulants are used both for the prevention and treatment of conditions like DVT and PE, and in the prevention of stroke in patients with atrial fibrillation [47]. Here is an overview of the key categories:

*Vitamin K Antagonists (VKAs):*
  - Warfarin: The most commonly used VKA, warfarin, works by inhibiting the synthesis of vitamin K-dependent clotting factors. Warfarin therapy requires the regular monitoring of the International Normalized Ratio (INR) to ensure therapeutic efficacy while minimizing the risk of bleeding. Its anticoagulant effect can be reversed with vitamin K administration, or prothrombin complex concentrate (PCC).

*Direct Oral Anticoagulants (DOACs):*
  - Dabigatran (Pradaxa^®^): A direct thrombin inhibitor. It directly inhibits thrombin (Factor IIa), preventing the conversion of fibrinogen to fibrin. Dabigatran offers the advantage of not requiring routine blood monitoring and has fewer food and drug interactions than warfarin. In patients with renal impairment, particularly those with severe impairment (e.g., creatinine clearance less than 30 mL/min), Pradaxa may not be used because it is primarily cleared by renal excretion.
  - Rivaroxaban (Xarelto^®^), Apixaban (Eliquis^®^), and Edoxaban (Savaysa^®^): These are direct Factor Xa inhibitors. They work by directly inhibiting Factor Xa, an important component in the coagulation cascade, thereby preventing thrombin formation and thrombus development. Similar to dabigatran, they have predictable pharmacokinetics and do not usually require routine monitoring.

*Advantages of DOACs over warfarin:*
  - Predictable anticoagulant effect, hence routine INR monitoring is not needed.
  - Fewer dietary restrictions.
  - Lower risk of certain types of bleeding, particularly intracranial hemorrhage.
  - Rapid onset of action.
  - Shorter half-lives, making them easier to stop before surgery or other procedures.

*Reversal of Anticoagulation:*
  - For warfarin: Vitamin K and PCC.
  - For dabigatran: Idarucizumab (Praxbind^®^) is a specific reversal agent.
  - For Xa inhibitors: Andexanet alfa (Andexxa^®^) can be used for reversal.

These anticoagulants are used in various settings for DVT prophylaxis, such as in patients undergoing major elective orthopedic surgery, those with prolonged immobilization, or in acutely ill medical patients. DOACs have many advantages. However, they are not suitable for all patients, such as those with mechanical heart valves or severe renal impairment. The choice of anticoagulant should be individualized based on the patient’s risk profile, renal function, and preferences. Furthermore, patient education on adherence, monitoring for bleeding, and interactions with other medications is vital in the management of patients on these therapies.

## 9. To Stop or Not to Stop Anticoagulation

Traditionally, the cessation of anticoagulation was influenced by the patient’s risk factors, type of spinal procedure, and bleeding risk. The timing for stopping anticoagulants before elective spine surgery is primarily determined by the drug’s pharmacokinetics and the patient’s renal function. For warfarin, it is generally recommended to discontinue 5 days before surgery to allow the normalization of the INR. Bridging therapy with LMWH may be considered for high-risk patients but is associated with an increased risk of bleeding. DOACs like rivaroxaban, apixaban, and dabigatran have shorter half-lives and are typically stopped 1–2 days before surgery depending on the patient’s renal function. The need for bridging therapy with DOACs is usually lower due to their rapid onset and offset of action. The timing for resuming anticoagulation after spine surgery was another critical decision often balancing the risk of thromboembolism and postoperative hematoma, which could be catastrophic in the spinal canal. Generally, anticoagulation can be resumed 24–48 h after surgery, provided hemostasis is achieved. However, this might be delayed in extensive open spinal surgeries with higher bleeding risks or if there are concerns about wound healing. In certain high-risk patients, such as those with a history of recent thromboembolic events, mechanical heart valves, or severe hypercoagulable states, the discontinuation of anticoagulation is riskier. In these cases, the risk of thrombosis outweighs the risk of surgical bleeding.

## 10. Spinal Surgery on Anticoagulation/Antiplatelet

For minor spine procedures or those with a low risk of bleeding, continuing anticoagulation might be considered, particularly in high-thrombotic-risk patients.

Since the type of spinal procedure, including the extent of surgical manipulation and the expected duration of the surgery, is a critical factor, procedures with minimal tissue disruption and low expected blood loss may be more amenable to the continuation of anticoagulation. It is the consensus of this team of authors, corroborated by years of clinical experience and evidence in the published literature [51,52], to continue anticoagulation for minor spinal procedures with low bleeding risk. This includes spinal endoscopic decompression surgery. High-risk patients, such as those with mechanical heart valves, recent thromboembolic events, or severe hypercoagulable states, also benefit from the continuation of anticoagulation (Figure 1).

**Figure 2:**
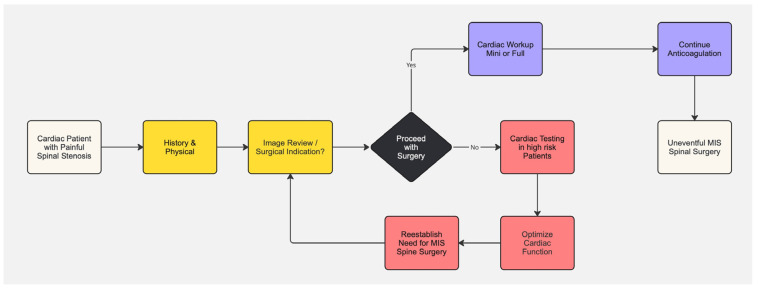
The flow chart summarizes the recommended management of anticoagulation and antiplatelet therapy in patients undergoing spinal procedures, focusing on those at different levels of surgical risk. Once the surgical indication is established and a surgery with minimal tissue disruption and low blood loss, such as spinal endoscopic decompression, is contemplated, the continuation of anticoagulation should be considered for minor procedures with low bleeding risk, particularly for those high-risk patients with mechanical heart valves, recent thromboembolic events, or severe hypercoagulable states. Elective minimally invasive spine procedures should be delayed at least one month after elective percutaneous coronary angioplasty, ideally six months. Procedures should be delayed 12 months after an acute coronary syndrome.

The same is considered for the antiplatelet agent management. It is the consensus of this team of authors, and recommendation from the published literature [2], to continue antiplatelet agents for minor spinal procedures with low bleeding risk. We recommend to delay elective minimally invasive spine procedures by a minimum of one month, and best until 6 months after elective percutaneous coronary angioplasty and 12 months after an acute coronary syndrome. The preoperative workup is summarized in the flow chart shown in Figure 2.

## 11. Drug Interactions with Antimicrobial Therapies

Although complications such as infections are uncommon, additional problems could arise in cardiac patients undergoing endoscopic spinal surgery, particularly those with pre-existing or new onset of atrial fibrillation. Therefore, the pharmacological interaction of anticoagulation and antiplatelet therapies with commonly used antibiotics must be considered [53,54,55,56,57,58,59]. These interactions may lead to enhanced anticoagulant effects due to antibiotic interaction, which can result in excessive bleeding and additional surgical and medical interventions. Reduced anticoagulant efficacy may also ensue, which can predispose patients to thromboembolic events, potentially leading to severe complications such as stroke [55].

Patients on warfarin therapy are particularly susceptible to interactions with antibiotics, which can affect the metabolism of warfarin and alter its anticoagulant effects. Antibiotics such as trimethoprim-sulfamethoxazole [57], metronidazole [60], and fluoroquinolones [59] can inhibit warfarin metabolism, leading to an increased risk of bleeding due to elevated INR (International Normalized Ratio) levels. Conversely, antibiotics like rifampin can induce hepatic enzymes, reducing warfarin’s effectiveness and increasing the risk of thromboembolic events. Direct oral anticoagulants (DOACs), including dabigatran, rivaroxaban, apixaban, and edoxaban, have fewer antibiotic interactions than warfarin [61]. However, certain antibiotics can still impact the pharmacokinetics of DOACs. For example, erythromycin and clarithromycin, which inhibit P-glycoprotein and CYP3A4, [53] can increase the plasma concentrations of DOACs, potentially raising the risk of bleeding. On the other hand, antibiotics like rifampin [55], which induce these pathways, can decrease the effectiveness of DOACs, heightening the risk of thromboembolic complications.

To mitigate these risks, several strategies should be considered. When antibiotics are necessary for treating postoperative infections, selecting those with minimal interaction with anticoagulants out of the ones to which the causative bacterium is susceptible is crucial. For instance, penicillins and cephalosporins typically have fewer interactions with anticoagulants and are often safer choices [54]. The close monitoring of INR levels is essential for patients on warfarin when antibiotics are prescribed [58]. Adjusting the warfarin dose based on INR readings can help maintain therapeutic levels and prevent complications. For patients on DOACs, monitoring for signs of bleeding or thrombosis and considering dose adjustments can help manage risks. A team-based approach between spinal surgeons, cardiologists, and infectious disease specialists should be employed to carefully coordinate all the aspects of the patient’s anticoagulation therapy and infection management.

## 12. Future Directions

Future research should prioritize refining preoperative cardiac evaluation protocols specifically tailored for ultra-minimally invasive endoscopic spine surgeries. Given the reduced surgical stress and lower risk profile of these procedures, updated guidelines are needed to balance the necessity of thorough cardiac assessments with minimizing unnecessary delays and interventions. Studies comparing the outcomes of streamlined versus traditional cardiac workups could provide valuable data to inform these new protocols.

The interplay between anticoagulation, antiplatelet therapies, and surgical outcomes highlights several critical areas where future research should be directed. For patients scheduled for ultra-minimally invasive endoscopic decompression surgery, comprehensive preoperative cardiac workups, as compared to traditional spine surgery patients, can lead to unnecessary system overutilization. Therefore, the authors propose that future research should aim to accomplish the following:

- Develop and Validate Streamlined Cardiac Risk Assessment Protocols**: Create protocols specifically designed for minimally invasive spinal surgeries.
- Compare Outcomes: Analyze the efficacy and safety of traditional versus simplified preoperative assessments to determine the necessity of reduced cardiac workups in this patient population.
- Correlate Protocols with Other Low-Risk Orthopedic Surgeries: Align new protocols with those in other low-risk outpatient orthopedic surgeries, such as hand surgery, to better predict cardiac risk for patients undergoing advanced spinal procedures.
- Evaluate Long-Term Outcomes and Quality of Life: Assess the long-term outcomes and quality of life in cardiac patients undergoing minimally invasive endoscopic spinal surgeries to identify any necessary protocol adjustments for addressing long-term clinical issues.

By addressing these research priorities, the outcomes for cardiac patients can be significantly improved, and healthcare delivery in this rapidly advancing field can be optimized.

## 13. Conclusions

Current ACC/AHA, ESC, and ASA guidelines emphasize individualized patient care. The changing views on the necessary preoperative workup for cardiac risk factors and anticoagulation management in patients undergoing elective minimally invasive and endoscopic lumbar spinal surgery underscores a pivotal shift in clinical practice. The traditional, one-size-fits-all approach is giving way to more nuanced and patient-specific strategies, reflecting the advancements in surgical techniques and a deeper understanding of the impact of perioperative care measures on clinical outcomes. Minimally invasive and endoscopic spinal procedures generally pose lower risks for significant bleeding and cardiac stress. Therefore, a more tailored approach to preoperative cardiac risk assessment, moving away from the extensive and often unnecessary testing that characterized traditional spine surgery protocols, is appropriate in low-risk patients. For patients with known cardiac disease or risk factors, the focus should be on optimizing existing conditions rather than extensive new evaluations, aligning with the latest ACC/AHA and ESC guidelines. While there is a general trend towards minimizing the interruption of anticoagulation in low-risk procedures, specific recommendations may vary depending on the clinical scenario. The decision to continue, modify, or discontinue anticoagulant therapy should be individualized, considering the patient’s thrombotic history, the type of anticoagulant used, and the specific nature of the spinal procedure. The shorter duration and less invasive nature of endoscopic surgeries offer an opportunity to minimize interruptions in anticoagulation therapy, reducing the risk of thromboembolic events while maintaining a low risk for surgical bleeding.

## Figures and Tables

**Table 1:** American College of Cardiology/American Heart Association (ACC/AHA) cardiac risk assessment criteria for patients undergoing non-cardiac surgery [3,4].

Risk Factor	Description
Major Criteria	Unstable coronary syndromes (e.g., unstable or severe angina, recent myocardial infarction) Decompensated heart failure Significant arrhythmias (e.g., high-grade AV block, symptomatic ventricular arrhythmias, supraventricular arrhythmias) Severe valvular disease
Intermediate Criteria	Mild angina pectoris Prior myocardial infarction Compensated or prior heart failure Diabetes mellitus (especially insulin-dependent) Renal insufficiency
Minor Criteria	Advanced age Abnormal ECG (e.g., LVH, left bundle-branch block, ST-T abnormalities) Rhythm other than sinus (e.g., atrial fibrillation) Low functional capacity (e.g., inability to climb one flight of stairs with a bag of groceries) History of stroke Uncontrolled hypertension
Surgical Risk	High: Cardiac risk often >5% (e.g., aortic and other major vascular surgery, peripheral vascular surgery) Intermediate: Cardiac risk generally 1–5% (e.g., intraperitoneal and intrathoracic surgery, orthopedic surgery) Low: Cardiac risk generally <1% (e.g., endoscopic procedures, superficial procedure, cataract surgery, breast surgery)

**Table 2:** American College of Cardiology/American Heart Association (ACC/AHA) guidelines for pre-operative echocardiogram published in 2014 [18].

Recommendation	Class	Level of evidence
Pre-op echo for valvular heart disease		
One year since last echo Change in clinical status since last evaluation	I	C
Echo to assess left ventricular function		
Dyspnea of unknown origin or h/o heart failure with worsening dyspnea	IIa	C
Stable CHF > 1 yr since last echo	IIb	C
Routine pre-op	III	B

## Data Availability

Not applicable.
